# *GRIA3*: proposal for a neuroimmune role linking infection, stress, chronic disorders and the activity of kynurenic acid

**DOI:** 10.3389/fncel.2026.1885761

**Published:** 2026-08-10

**Authors:** Trevor W. Stone, Felix I. L. Clanchy, Richard O. Williams

**Affiliations:** Nuffield Department of Orthopaedics, Rheumatology and Musculoskeletal Disorders, The Kennedy Institute of Rheumatology, University of Oxford, Oxford, United Kingdom

**Keywords:** AMPA receptors, chronic disorders, epilepsy, *GRIA3*: the gene producing protein GluA3, kynurenic acid, neuroimmune interface, plasmacytoid dendritic cells, schizophrenia

## Abstract

Glutamate receptors in the CNS sensitive to the analog AMPA regulate neuronal excitability and synaptic plasticity. AMPA receptors are usually hetero-tetramers and the human *GRIA3* gene and GluA3 protein product have been implicated in various CNS abnormalities including epilepsy, multiple sclerosis, schizophrenia, and cognitive disorders. Although not generally found in the immune system, *GRIA3* has recently been demonstrated in a highly specialized group of immune system cells–plasmacytoid dendritic cells (pDCs). These are among the first cells to be activated by infecting viruses and they express *GRIA3* in the absence of other AMPA receptor subunits, suggesting a possible role for the subunit in those neuroimmune disorders. The potential for molecular interactions between pDCs and neurons or glia is discussed, with the implications for linking viral infections, pDC activation, and chronic diseases. With growing evidence that the tryptophan metabolite kynurenic acid—an AMPA antagonist—is involved with a similar range of disorders, its role in those links is also discussed.

## 1 Introduction

Many disorders of the major organ systems and Central Nervous System (CNS) are considered to begin with single, brief episodes of disturbance such as a head trauma or an episode of infection, especially if experienced at a vulnerable time such as pregnancy. It has been difficult to understand the chronic nature of these sequelae without recourse to some form of long-term genetic modification. The recent discovery of a key glutamate receptor subunit in virus-activated cells of the immune system raises the possibility of explaining these disorders by close interactions and macromolecule transfer between cells of the immune system and CNS.

## 2 AMPA receptors for glutamate in the CNS

Glutamate and its ionotropic receptors responding to the structural analogs kainic acid or α-amino-3-hydroxy-5-methyl-isoxazole-4-propionic acid (AMPA) are responsible for

the fast depolarization produced by glutamate released at neuronal synapses (Baranovic, 2021; de Leon-López et al., 2025; Diering and Huganir, 2018; Henley and Wilkinson, 2016; Huganir and Nicoll, 2013; Sun et al., 2008). The family of N-methyl-D-aspartate (NMDA)-sensitive receptors generate a slower, more prolonged depolarization which, together with the fast AMPA receptor (AMPAR) activity, are involved in many aspects of neuronal plasticity, learning and cognition. Agonist ligands are of value for disorders involving neural hypo-activity such as cognitive dysfunction, while antagonists reduce the hyper-excitability of seizure disorders (Leo et al., 2018; Perversi et al., 2023). Both types of ligand can limit the effects of neurodegeneration, since agonists improve the declining network activity and antagonists prevent damage to neurons and glia (Rektor, 2013; Russo et al., 2012; da Silva and Schroeder, 2023; Ning et al., 2024).

### 2.1 GRIA3 and GluA3 in the CNS

AMPAR are normally hetero-tetrameric complexes of protein subunits GluA1-GluA4 (genes *GRIA1-4*). Neurons expressing different combinations of these subunits exhibit marked differences in their electrical properties, including their sensitivity to glutamate, time course of onset and duration of depolarization and repolarization, and the time course of desensitization and re-sensitization (Jacob and Weinberg, 2015; van der Spek et al., 2022). The deletion of GluA3 in particular reduces neural excitability (Reinders et al., 2016; Peng et al., 2022), reduces decay times and impairs neuro-transmission (Antunes et al., 2020). Receptor composition also affects cellular trafficking and localization of complete receptors, with profound effects on neural activity (de Leon-López et al., 2025; Ge and Wang, 2021; Henley and Wilkinson, 2016; Shepherd and Huganir, 2007; Diering and Huganir, 2018). However, with this range of properties in the CNS, the *GRIA3* gene and its GluA3 subunit protein product have been specifically implicated in many disorders such as those listed in Table 1.

**Table 1:** Physiological and pathological involvement of the *GRIA3*-GluA3 axis.

*GRIA3*-GluA3 modification	Biomedical effects	References
*GRIA3* interacts with Xpo7 protein	regulates cyclic AMP sensitivity; linked to schizophrenia	Toyoda et al., 2025
GluA3 expression	chronic presence of β-amyloid reduced expression	Prinkey et al., 2024
*GRIA3* variant	neurodevelopmental changes	Rinaldi et al., 2024
GluA3 dependent effects	β-amyloid inhibition of spine formation and synaptic transmission	Zhang et al., 2023; Reinders et al., 2016
GluA3 expression	reduced in patients with Alzheimer's disease	Zhang et al., 2023; Reinders et al., 2016
*GRIA3* variant - pGlu787Lys'	encephalopathy and epilepsy	Melnikova et al., 2022
*GRIA3* mutations	linked to epilepsy^*^ consistent with Bonnet et al., 2009	Rinaldi et al., 2022
GluA3 deficiency	reduced glutamatergic function and increased aggression	Peng et al., 2022
GluA3	interaction with miRNA-212- p5 linked to lipid accumulation	Hu et al., 2022
*GRIA3* polymorphism	Implicated in seizures, autism, schizophrenia	Singh et al., 2022
*GRIA3* expression	linked with amphetamine dependence	Iamjan et al., 2018
GluA3 expression	changes in monocytes during pregnancy	Bhandage et al., 2017
*GRIA3* expression	linked to migraines	Maher et al., 2013
GluA3 loss	increased striatal [DAm] with enhanced social interaction and aggression	Adamczyk et al., 2012
GluA3 expression	increased in CSF of Lewy-Body Disease	Enache et al., 2020
*GRIA3* mutation	reduced AMPAR desensitization and increased glutamatergic transmission leading to musculoskeletal dysfunction	Sun et al., 2021; Hamanaka, 2022; Piard et al., 2020
*GRIA3* mutations	Seizures produced	Malina et al., 2006; Allen et al., 2021; Okano et al., 2023; Rinaldi et al., 2022, 2024; Necpál et al., 2023
GluA3 antibodies	linked to epilepsy^*^	Scheggia et al., 2021
GluA3 deletion	affects sleep quality	Davies et al., 2017; Volk et al., 2018
GluA3 deletion	inhibited cerebellar function	Gutierrez-Castellanos et al., 2017
GluA3 Ab	associated with tau phosphorylation and cognitive performance	Palese et al., 2020; Benussi et al., 2019; Borroni et al., 2017; Italia et al., 2021, 2022, 2024
*GRIA3*- GluA3 deletion	inhibits synaptic plasticity and learning	Wang et al., 2011; Meng et al., 2003; Reinders et al., 2016; Peng et al., 2022
*GRIA3* expression	correlated with cognitive decline in Alzheimer's but not Parkinson's or Lewy Body dementia	Bereczki et al., 2018
*GRIA3* interactions	induces flop variants of other subunits, producing faster kinetics, transmission decay and desensitization;—deletion reduces decay time	Antunes et al., 2020
GluA3 expression and activity	expression increased in models of Alzheimer's disease, but β-amyloid inhibits activity	Renner et al., 2017; Jia and Collingridge, 2017; Milham et al., 2024
*GRIA3* loss in cingulate cortex	X-linked mental retardation	Wu et al., 2007; Gecz et al., 1999; Chiyonobu et al., 2007
*GRIA3*- silencing mutation	epilepsy and intellectual disability	Bonnet et al., 2009
GluA3 deletion	linked to Autism Spectrum Disorder	Guilmatre et al., 2009
GluA3 deletion	reduced synaptic efficacy in amygdala	Humeau et al., 2007
GluA3 antibodies	Rasmussen's encephalitis (includes frequent seizures, paralysis and cognitive dysfunction)	Rogers et al., 1994

Examples of the relationship between GRIA3-GluA3 and clinical disorders.

^*^A role for AMPARs in seizures is supported by use of anticonvulsant drugs such as perampanel and talampanel, which are selective antagonists on AMPARs (Yuan et al., 2019).

### 2.2 Structural features of AMPARs

AMPAR subunits have usually been found naturally in combination with at least one other subunit, even though individual subunits may influence only some aspects of the full receptor activity. In the complex of GluA2 and GluA3, for example, it is GluA3 that has the dominant and significant influence on synaptic speed, promoting synaptic vesicle release and plasticity (Antunes et al., 2020). Increased levels of cyclic AMP increase the conductance specifically of GluA3-operated channels leading to a potentiation of transmission (Renner et al., 2017). In developing brain, thalamic neurotransmission is mediated almost exclusively by AMPAR composed only of GluA3 and GluA4 subunits (Wang et al., 2011) but only the deletion of GluA3 affected learning-induced synaptic plasticity (Reinders et al., 2016; Meng et al., 2003).

All four AMPAR subunits (GluA1-4) can assemble into homomeric or heteromeric receptors when expressed in appropriate cells (Traynelis et al., 2010; Szymanska et al., 2017a,b; Coleman et al., 2010, 2016; Nakanishi et al., 1990; Wenthold et al., 1996; Ge and Wang, 2021) and all those combinations respond to glutamate, the natural ligand (Banke et al., 1997; Kristensen et al., 2011).

### 2.3 GRIA3 and GluA3 in the immune system

Although GluA3 is primarily expressed by neurons and glia in the CNS, a recent study of all the glutamate receptors found that *GRIA3* was expressed at a relatively high level in a small population of highly specialized leucocytes in the immune system—the plasmacytoid dendritic cells (pDCs) (Clanchy et al., 2026). Of the many varieties of leucocytes in the immune system, pDCs exhibit a special relationship with the *GRIA3*-GluA3 axis. The cells comprise < 0.5% of the total leucocyte population in humans. They can arise from common lymphoid or myeloid progenitors (Facchetti et al., 1988a), with properties typical of classical DCs (cDCs) but expressing a marker profile which includes CD74 (the MHC-Class II invariant chain), CD123, CD303, CD304 (Facchetti et al., 1988b) and interleukin-7-receptor (IL7R) (Rodrigues and Tussiwand, 2020). Unlike cDCs, they are found mainly in the blood and lymphoid organs (Facchetti et al., 1988b), with unique features not found in cDCs (Adams et al., 2024). Three subsets of pDCs have been recognized in pDCs activated by influenza infection, the transcript for *GRIA3* being modestly higher in the P3 pDC subset (PD-L1-CD80+) compared to the P1 (PD-L1+CD80-) subset with the P2 subset (PD-L1+CD80+) having intermediate expression (Alculumbre et al., 2018).

Although pDCs do not proliferate directly, activation promotes their maturation to cDC-like cells which do proliferate (Soumelis and Liu, 2006). More pDCs are then produced *de novo* so that their overall number remains unchanged or increases relative to the normal steady state. Functionally, pDCs are considered to bridge the processes of innate and adaptive immunity (Li et al., 2017), with a significant influence on autoimmune disorders such as Systemic Lupus Erythematosus (SLE) and rheumatoid arthritis (Colonna et al., 2004; Swiecki and Colonna, 2010; Jegalian et al., 2009). After activation their differentiation to cDCs leads to an expansion of anti-inflammatory regulatory T cells (Stone and Williams, 2023), but a major part of their functional importance is that they are the first cells to detect viral infections (Cella et al., 2000).

### 2.4 pDC activation by viruses

Overall, pDCs are crucial regulators of autoimmune and allergic reactions (Takagi et al., 2011; Villadangos and Young, 2008; Adams et al., 2024; Colonna et al., 2004; Rowland et al., 2014). The presence of viral nucleic acid fragments in pDCs activates intracellular Toll-Like Receptors (TLR), mainly TLR7 and TLR9. Viral exposure induces pDCs to differentiate into cDC-like cells and, under the chemoattractive influence of the chemokine CCR7, to migrate to the blood and lymphoid organs where they modulate T cell development. The maturation of pDCs is accompanied by the expression of MHC Class I and II molecules necessary for interactions with both CD8+ and CD4+ T cells, respectively (Villadangos and Young, 2008; Jegalian et al., 2009). The immediate response to viral stimulation is to generate type I interferons (IFNα- and β-) which then induce IFN-γ in macrophages and Natural Killer (NK) cells. They also express large quantities of pro-inflammatory cytokines such as Tumor Necrosis Factor-α (TNF-α) (Wacleche et al., 2018; Reizis, 2019) along with modulators of T cell activity which limit inflammation in tissues including the CNS (Mundt et al., 2019; Giles et al., 2018; Stone and Williams, 2023).

### 2.5 GluA3 “flip” in pDCs

A remarkable feature of the *GRIA3* gene in pDCs is that it is expressed alone, in the absence of any other AMPAR subunits (Clanchy et al., 2026). These subunits will be available in their singlet form but may also be able to form stable homomeric receptors and behave as fully functional AMPARs as discussed below.

In neurons, GluA3 can exist in two molecular conformations known as “flip” and “flop,” with the “flop” molecules exhibiting greater conformational flexibility, reducing channel open time and the rate of desensitization (Pei et al., 2007, 2009). Developmental or activity-dependent changes in the ratio between the variants is a recognized mechanism of feedback control of excitability and plasticity which is still observed when the isoforms are expressed in a range of cell types (Nishimura et al., 2000; Akinshola et al., 2003; Wang et al., 2016). Although GluA3 in pDCs is entirely in the ‘flip' conformation (Clanchy et al., 2026) its presence in cells expressing other subunits promotes their insertion as the “flop” forms. This causes synaptic potentials to exhibit faster kinetics with more rapid decay and desensitization times which affect behavioral cognitive performance. These contributions of GluA3 to the regulation of plasticity and cognition is dependent on cyclic AMP, probably because of its direct association with “Exchange Protein directly Activated by cyclic AMP-2” (Epac2) (Zhang et al., 2024; Renner et al., 2017). In addition, there are significant pharmacological differences between the two variants (Sekiguchi et al., 2002; Schmid et al., 2001; Stine et al., 2001). The relative amounts of “flip” and “flop,” in the CNS, for example, are region-dependent, with up to 3-fold more flop than flip in the CA1 hippocampus, but 3-fold more flip than flop in the CA3 region. These levels can be affected by a range of drugs such as opiates (Park et al., 2003).

## 3 *GRIA3* and GluA3 in clinical disorders

A fuller understanding of the *GRIA3*-GluA3 axis is required in view of the wide range of medical conditions in which the combination has been implicated (Table 1). Polymorphisms have been described, especially in subjects with intellectual disabilities, when gene sequencing revealed several missense variants of *GRIA3*, at least four of which were located in the GluA3 functional domains (Moretto et al., 2018). A total loss of the whole gene was also observed in some cases. An examination of all four subunits identified 32 mutations in the *GRIA3* gene, all of which produced changes in response sensitivity, magnitude and time course of cellular responses (XiangWei et al., 2023).

The concept of immune system involvement in CNS function and disease was strengthened by evidence that CD4+ T cells were required for individuals to respond positively to an otherwise neuro-degenerative situation (Kipnis et al., 2004; Kipnis, 2016; Ron-Harel and Schwartz, 2009). Depletion of those cells depressed cognitive function and associated behaviors, which were normalized by the adoptive transfer of fresh T cells. It is possible that the age-related decline in cognitive ability may also be related to impaired adaptive immunity (Ron-Harel and Schwartz, 2009).

While much of the evidence arose from experimental studies, observations of human subjects have confirmed that maternal infection or inflammation during pregnancy significantly increases the risk of behavioral abnormalities in the offspring, most notably those seen in schizophrenia (Brown, 2011a,b; Brown and Patterson, 2011; Girchenko et al., 2020). Viral infections seem to be particularly involved in the initiation of chronic disorders (Yates and Mulkey, 2024). Indeed, the problem of Infection-Associated Chronic Illnesses (IACI) is increasingly being recognized (Hernandez et al., 2026). Maternal stress also affects development of the embryos and neonates (Brown, 2011a; Meyer et al., 2008; Meyer and Feldon, 2010) and several psychiatric and neurological disorders are considered to be in this category of “developmental disorders” (Meyer and Feldon, 2010; Hornig et al., 2018; Pearce, 2003; Brown, 2011a). Glutamate-releasing neurons have been associated with many of these (Moretto et al., 2018), with schizophrenia in particular being considered to be primarily a result of reduced glutamatergic activity (MacDonald et al., 2015; Tamminga et al., 2012; Stan et al., 2015) and for which agonists for AMPARs (AMPAkines) have been developed for treatment (Lynch and Gall, 2006; Henry et al., 2026). It is potentially highly relevant, therefore, that mutations in the *GRIA3*-GluA3 axis have been linked with a variety of CNS neurological and psychiatric conditions (Table 1) including schizophrenia. As a pathway able to modulate glutamate receptor function, the metabolism of tryptophan to kynurenine and related compounds may be involved in some cases, as discussed later.

## 4 Proposal for *GRIA3* as a mediator of neuroimmune communication by intercellular transfer

**Figure 1:**
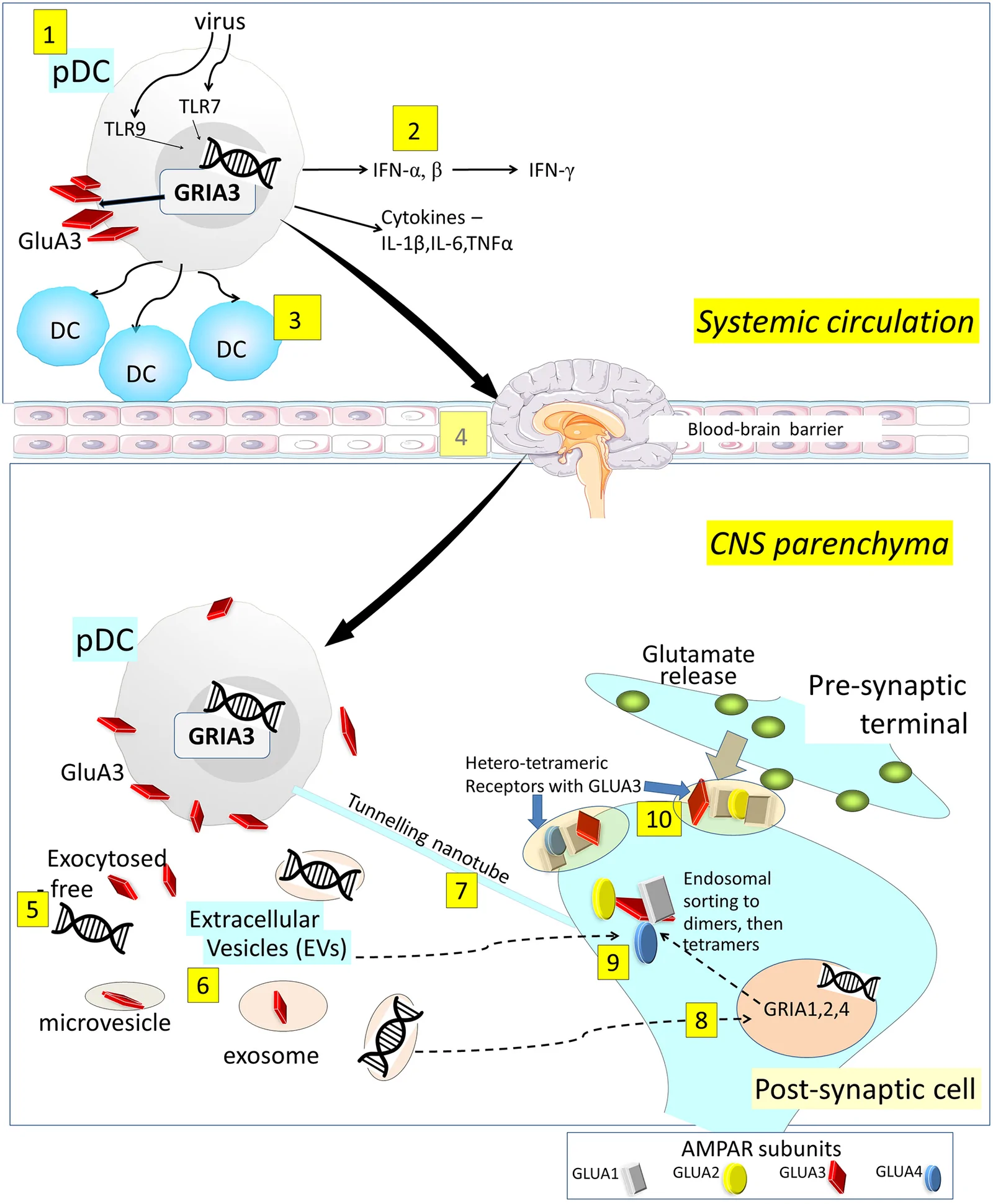
Route from plasmacytoid cells to the CNS. A summary of the proposed steps linking viral infection to chronic changes in the CNS. (1) Plasmacytoid cells (pDCs) express *GRIA3*. TLR7 and TLR9 receptors are among the earliest to responding to viral nucleic acid fragments. (2) TLR activation induces production of Type 1 interferons (IFN-α and -β) which initiate the host immune system response and cytokine release. (3) The pDCs differentiate to cDCs which proliferate to be replaced by newly generated pDCs. (4) The penetration of pDCs into the CNS is increased by chemoattractive cytokines increased blood-brain barrier permeability. (5) *GRIA3* gene (or GluA3 protein) could be transferred from pDCs to neurons and glia as free molecules or (6) carried by Extracellular Vesicles and (7) nanotubes. (8) After endocytosis, *GRIA3* would be translated to GluA3 for passage to the endosomal compartment (9) for sorting, complex formation if necessary and insertion into the cell membrane (10). Steps indicated by solid arrows are supported by evidence relating to most AMPAR subunits; the more speculative links in this proposal (6–10) are shown with dashed arrows.

Given the number of medical conditions in which GluA3 has been implicated, the question arises of whether there is any link between the expression of GluA3 alone or as homomers in pDCs and the etiology of those disorders. In particular, the role of pDCs as the initial sensors of viral infection and the subsequent recruitment of other cell groups in the immune system suggests that there might be a mechanism by which activated pDCs could modify cell properties in systemic tissues and the CNS. Two factors would be required for this—the presence of pDCs in the tissues and CNS, and a means of communication between pDCs and other cells including neurons and glia. We propose that the virally-induced activation of pDCs increases their numbers in the CNS and that *GRIA3* or GluA3 will transfer from them into neurons or glia. The expression of GluA3 in these cells, as well as resident neurons, could potentiate neuronal responses to the combined influences of neuroglial and inflammatory ligands. While speculative, the additional transfer of *GRIA3* or GluA3 (possibly both) from activated pDCs to CNS neurons and glia would then increase GluA3 expression and could produce long-lasting changes in the subunit balance leading to altered neuronal excitability and plasticity. Such changes in neuroglial activity could then contribute to chronic clinical disorders such as epileptic syndromes, schizophrenia and other symptoms of cognitive dysfunction (Table 1; Figure 1). This concept might include the transfer of mutant nucleic acids generated peripherally but then transferred to neuroglia, disseminating any mal-functioning activity to the CNS. It would also be more important when RNA, or single- or double-stranded DNA has been transferred between cells in extracellular vesicles (Rai et al., 2021a,b; O'Brien et al., 2020; Ekström et al., 2012; Payandeh et al., 2024).

In the following sections, we address each of the features and processes that would be necessary for this proposal, indicating how existing evidence on *GRIA3* and other AMPAR subunits provide significant support for the concept.

### 4.1 pDCs in the CNS

Although there are few DCs present in the normal, uninjured CNS parenchyma, they do enter the brain if there is local damage from an injury or stroke, or during peripheral inflammation, infections or exposure to stress (Serafini et al., 2006; Zozulya et al., 2010; Yogev et al., 2012; Hoye et al., 2018). Following an episode of cerebral ischaemia, activated microglia attract the movement of DCs into the brain parenchyma, including cDCs and pDCs (Gallizioli et al., 2020). The trafficking of pDCs into the CNS is regulated partly by microRNA species (Hoye et al., 2018) and chemokines such as CCL17 (Ruland et al., 2017), with recruitment enhanced in the early stages of the multiple sclerosis model Experimental Allergic Encephalomyelitis (EAE) (Sie et al., 2019). Cytoskeletal changes are also involved (Meena et al., 2021).

The role of pDCs may then become pivotal since the IFN-β produced by them will contribute to the further influx of cDCs (Pennell and Fish, 2017) and their promotion of regulatory T-cells (Tregs) differentiation and immune tolerance. Similarly, the expression of MHC (Major Histocompatibility Complex) proteins and T cell activation modulators such as CD80 and CCR7 persists for long periods after inflammation (Bossu et al., 2015; Clarkson et al., 2014). Increased pDC penetration to the CNS is seen in the early stages of autoimmune disorders such as MS / EAE, when they contribute to the suppression of local CD4+ T cells and reduce the severity of disease (Duraes et al., 2016; Bailey et al., 2007; Giles et al., 2018; Mundt et al., 2019). Conversely, their depletion increases central CD4+ activation and IFN-γ generation, exacerbating the condition (Bailey-Bucktrout et al., 2008; Colonna et al., 2004).

An additional consideration of immune system cells in the CNS is that following infections, systemic inflammation or cerebral incidents such as vascular infarcts and the resulting higher rates of cell death, there will be an increased permeability of the blood-brain barrier, allowing increased movements of cells and molecules between immune system cells and the CNS (Figure 1). This could represent one explanation of the development of neuro-psychiatric disorders in the aftermath of physical trauma, infection or chronic stress, for example, which would further increase the cell and molecular movements described above.

### 4.2 GluA3 receptors in pDCs

The intercellular movement of AMPAR subunits or intact tetrameric receptors has been observed, with efficient transfers of each of the subunits (Peters et al., 2021; Ge and Wang, 2021; Zachariassen et al., 2016; Rinaldi et al., 2024; Hennegriff et al., 1997). All AMPAR subunits can form homomeric receptors with electrophysiological activity in sensitive patch clamp studies (Sudo et al., 1997, 1999), while their combined transfection—for instance GluA1 and GluA2—produces heteromeric combinations of the same stoichiometry as natural GluA1/GluA2 receptors (Nishimura et al., 2000; Ge and Wang, 2021). This organization may be related to the continuous recycling of the composite mixtures between their endosomal location and the postsynaptic membrane. These receptors are trafficked to the cell membrane and exhibit functional activity comparable with the natural receptors (Rinaldi et al., 2024; Toyoda et al., 2007). With neurons, the expression of physiological functionality extends to their electrophysiological properties (Neve et al., 1997) and AMPAR subunits transfected into hippocampal neurons by viral transfer produce functional receptor combinations in intact multi-neuronal circuits (Kakegawa et al., 2004).

Clearly, the presence of *GRIA3* alone in pDCs precludes the formation of heteromeric receptors, but the translation of *GRIA3* RNA into the GluA3 (flip) protein has been reported many times (Varney et al., 1998; Poon et al., 2010; Pokharna et al., 2025; Banke et al., 1997; Holley et al., 2012; Yuan et al., 2019; Man et al., 2000a,b; Limon et al., 2007; Akinshola et al., 2003; Ge and Wang, 2021; Zachariassen et al., 2016; Hennegriff et al., 1997). Homomeric GluA3 receptors behave as fully functional AMPARs, being activated by glutamate and blocked by the general quinoxaline-based AMPA antagonists such as CNQX and NBQX (Tondreau et al., 2008; Holley et al., 2012; Fukushima et al., 2020) as well as the AMPA-selective anticonvulsant drug perampanel (Yuan et al., 2019). An important feature of GluA3 is that its molecular structural flexibility predisposes it to interact preferentially with other AMPAR subunits (Sukumaran et al., 2011; Coleman et al., 2010; Zhao et al., 2016). As a result, GluA3 homomers can only form in significant numbers in the absence of other subunits (Greger et al., 2017; Hansen et al., 2021; Coleman et al., 2006, 2010). This makes the presence of homomeric GluA3 complexes in pDCs easier to understand, as no other subunits are present. The significance of this will be addressed later.

However, it should be noted that the format of GluA3 expression and the question of functionality are not a central issue in the current proposal. Multi-unit receptors may be involved where functional responses have been obtained, since the individual subunits would probably not be functionally competent, but this not would be required for our proposal: pDCs expressing only singlet subunits would still be able, in principle, to transfer these to other cells using the various mechanisms discussed below. Our proposal is predicated only on the viral activation of pDCs and their transference of *GRIA3* or GluA3 to cells where its influence will affect activity.

### 4.3 Intercellular molecular transfer

The fundamental importance of the intercellular transfer of proteins, RNA and DNA molecules has been recognized for more than 25 years (Valadi et al., 2007; Davis, 2007; Armingol et al., 2021; Lanna et al., 2022; Simons and Raposo, 2009; Diehl et al., 2008). The nuclear machinery and subsequent endosomal sorting produces functional receptor complexes of the same multimeric subunit combinations that occur naturally in similar populations of the recipient cells as noted above for GluA1/GluA2 and GluA3/GluA4 combinations. The process of intercellular transfer is sometimes referred to as “lateral (or horizontal) gene transfer” by analogy with the process originally demonstrated in prokaryotes and essential for environmental adaptation and antimicrobial resistance (Keeling and Palmer, 2008; Chen et al., 2012; Soucy et al., 2015; Emamalipour et al., 2020). The process is considered physiologically important in many situations such as neuronal network integration and for the development of tumor resistance (Valcz et al., 2022) (Figure 1).

The fidelity of the transfected cell properties compared with natural cells has been confirmed by the selectivity and potency of antagonists (Nielsen et al., 1998).

For pDCs and other immune system cells, the most likely mechanism of interaction is the movement of molecules between cells. In addition to the very rapid, millisecond duration, changes of excitability mediated via synaptic transmission, cells in the CNS—and most other tissues—also exchange molecular information on slower time scales. This can include the movement of molecules from one cell to another via the intercellular fluid. All extracellular (inter-cellular) body fluids contain a variety of macromolecules, including circulating cell free (cf)RNA or cfDNA, and proteins including many enzymes (Stewart and Tsui, 2018; Yao et al., 2016; Thierry et al., 2016; Tamkovich et al., 2006; Jiang and Lo, 2016; Sun et al., 2015; Lehmann-Werman et al., 2016). They are often the result of cell damage or death but, since ongoing cell death is a feature of all living organisms, they are present in normal, as well as ill, populations (Yeri et al., 2017; Zhou et al., 2021, 2022). The principle of intercellular transfers of such macromolecules between immune system cells has become well established (Groc and Choquet, 2006). From the extracellular medium, these molecules can be exchanged between cells via the common processes of exocytosis and endocytosis, both of which have been demonstrated to occur with AMPAR subunits (Hirano, 2018). The differential secretion by exocytosis of proteins and nucleic acids, followed by their endocytosis into other cells, has been specifically proposed as an important factor in the frequently observed mismatch between gene and protein expression in many cells (Jiang et al., 2020).

These molecular movements may involve diffusion or active transport of the molecules themselves, although many macromolecules can be ferried between cells as cargo in cell organelles and extracellular vesicles (Figure 1) (Nevarez-Ramirez et al., 2023; Thierry et al., 2016; Yeri et al., 2017; Yu et al., 2015; Yang et al., 2017; Shao et al., 2015; Huang et al., 2013; Li et al., 2014; Lunavat et al., 2015). The general term Extracellular Vesicles (EVs) includes structures such as microvesicles and exosomes with a range of sizes from 20–500 nm (Raza et al., 2016; Cocucci and Meldolesi, 2015) although the description and definition of these is in need of clarification (Torres and Lee, 2023). The particular importance of EVs arises partly from their ability to transfer a wide range of molecular “cargoes” of proteins and genetic material, including RNA and single- or double-stranded DNA (Rai et al., 2021a,b; O'Brien et al., 2020; Ekström et al., 2012; Payandeh et al., 2024). The movement of molecules by EVs does result in functional changes in the recipient cells' behavior and fundamental properties such as apoptosis and metastasis (Thery et al., 2009). Neurons secrete and absorb EVs with effects on axonal structure and the formation or pruning of synapses in addition to excitability and plasticity (Torres and Lee, 2023; Raza et al., 2016). EVs may also transfer mutated molecules, thus effecting a form of epigenetic, instant evolution on the tissue system concerned (Jahan et al., 2022).

Related to the vesicles are “nanotubes,” filamentous structures commonly found in the CNS physically bridging the space between neurons and glial cells (Figure 1). These are relatively large structures—around 50 μm wide and 50–500 μm long, although even larger ones have been reported. They conduct a wide variety of molecules and can even conduct organelles including mitochondria (Agnati et al., 2010; Agnati and Fuxe, 2014; Jahan et al., 2022).

Molecular transfer can also include trogocytosis—the absorption of part of a cell that has been described in many cases including T and B lymphocytes, Natural Killer cells and antigen presenting cells (Brown et al., 2012a,b). Very large molecules such as antigenic proteins, MHC proteins and their receptors can be conveyed in this fashion (Zhao et al., 2022; Reed et al., 2021; Nakayama et al., 2021; Miyake and Karasuyama, 2021).

These various processes can transport a wide variety of molecules, small or large, such as nucleic acids or fragments thereof, in addition to proteins, lipids and other compounds (Charreau, 2021) including glutamate receptors (Newpher and Ehlers, 2008). Both GluA1 and GluA3 subunits can be trafficked in this way, although a difference in the sizes of vesicles carrying them suggested a functional mechanism to specify different cell or membrane localizations of the two molecules (Peters et al., 2021). Experimental confirmation that complete genes for receptors exchange between cells and the extracellular medium is exemplified by the demonstration of the presence of glutamate receptor genes in human blood serum and exosomes (Kamyshna et al., 2022; Sanchez-Melgar et al., 2020).

Importantly, the ability of extracellular nucleic acids to enter cells and to integrate into their genome would account for the long-lasting nature of chronic disorders including neurological conditions (Parkinson's disease, schizophrenia, many disorders involving deficits in cognition and mental development) resulting from alterations in AMPAR composition. Those abnormalities would probably be irreversible in view of the high affinity of GluA3 for all other AMPAR subunits.

Indeed, intercellular transfer of the *GRIA3* gene would probably integrate into the recipient cells' genomes, causing effects that might be irreversible and require the development of pharmaco-genetic strategies for cure rather than conventional approaches focused on symptom control. Such genetic integration has been demonstrated for a range of molecules including the transfer of intact telomeres from antigen presenting cells (Lanna et al., 2022).

### 4.4 Transfer between cell types

Importantly for the present discussion, such molecular movements can occur between most types of leucocytes, often of molecules with immunological relevance such as MHC proteins (Zhao et al., 2022). EVs have been identified as a major mechanism for the transfer of genes between leucocytes and CNS neurons and glia (Ridder et al., 2014; Deng et al., 2022). They also carry macromolecules between cells in peripheral organs and tissues to leucocytes (Nevarez-Ramirez et al., 2023) and to neurons or glia (Nishiyama et al., 2016), exemplified by RNA transfer between glia and macrophages (Paolicelli et al., 2019). Within the CNS, RNA transfer has been considered crucial to interneuronal integration (Meservey et al., 2021; Zappulli et al., 2016) and has been studied for its contribution to transfers between neurons to leucocytes and *vice versa* in relation to the genesis of Alzheimer's disease (Deng et al., 2022) (Figure 1).

AMPAR subunits have been shown to transfer between all cell types in the CNS by exocytosis and endocytosis (Hirano, 2018). However, as noted above, the unique flexibility of the GluA3 structure leads it to interact preferentially with the other three AMPAR subunits (Coleman et al., 2016). For those pDCs which enter the CNS, therefore, there would be a strong tendency for GluA3 subunits to leave the pDCs and transfer into neurons expressing AMPARs which do not include GluA3. This would apply most to singlet GluA3 subunits, but the greater affinity of GluA3 for other AMPAR subunits would result in any homomers present in pDCs being relatively unstable and ready to “donate” (or lose) individual GluA3 subunits to existing heteromeric complexes in CNS cells. The shift in AMPAR composition would cause a significant change in the electrical and metabolic properties of the recipient cells as noted earlier. Effectively, a bias would develop for the transfer of GluA3 from pDCs to neurons and glia which express those other subunits. The pDCs would be acting as a source or reservoir of GluA3, whereas cells with different subunits would attract GluA3 to form functional GluA3-containing heteromers.

Adding to these forces is that the “flip” variants of AMPAR subunits in pDCs can be actively secreted by cells, whereas the “flop” isoform tends to be retained intracellularly, mainly associated with endoplasmic reticulum (Coleman et al., 2006). Since the GluA3 isoform in pDCs is entirely in the “flip” variety (Clanchy et al., 2026), the subunits are more likely to be shed than other subunits and hence more amenable to transfer into cells expressing other AMPAR subunits where they would significantly alter cell properties.

These consideration are especially relevant to the presence of *GRIA3*-GluA3 expression as mediators of neuroimmune communication in the CNS and immune system cells, since the higher the proportion of GluA3 in the glutamate receptor pool, the more diluted will be the normal multi-unit heteromeric subunits. Since each subunit is linked with specific cell properties, this would change the balance of AMPAR function as a whole. For example, GluA2 is a key factor regulating calcium permeability, while GluA1 has been linked with novelty recognition behavior, learning, and memory (Meng et al., 2003). Their dilution resulting from cells acquiring GluA3 would alter these aspects of physiology indirectly, in addition to the properties introduced by GluA3.

Of course, some molecular transfers may be bidirectional, with some movements occurring from neurons and glia into immune system cells, potentially impacting on immune system function. Such a bi-directional interchange would contribute substantially to the recognized reciprocal influences of neuroimmune communication known as the neuroimmune interface (Zouikr et al., 2017; Stone et al., 2022; Stone and Williams, 2024).

## 5 A key role for the kynurenine pathway

**Figure 2:**
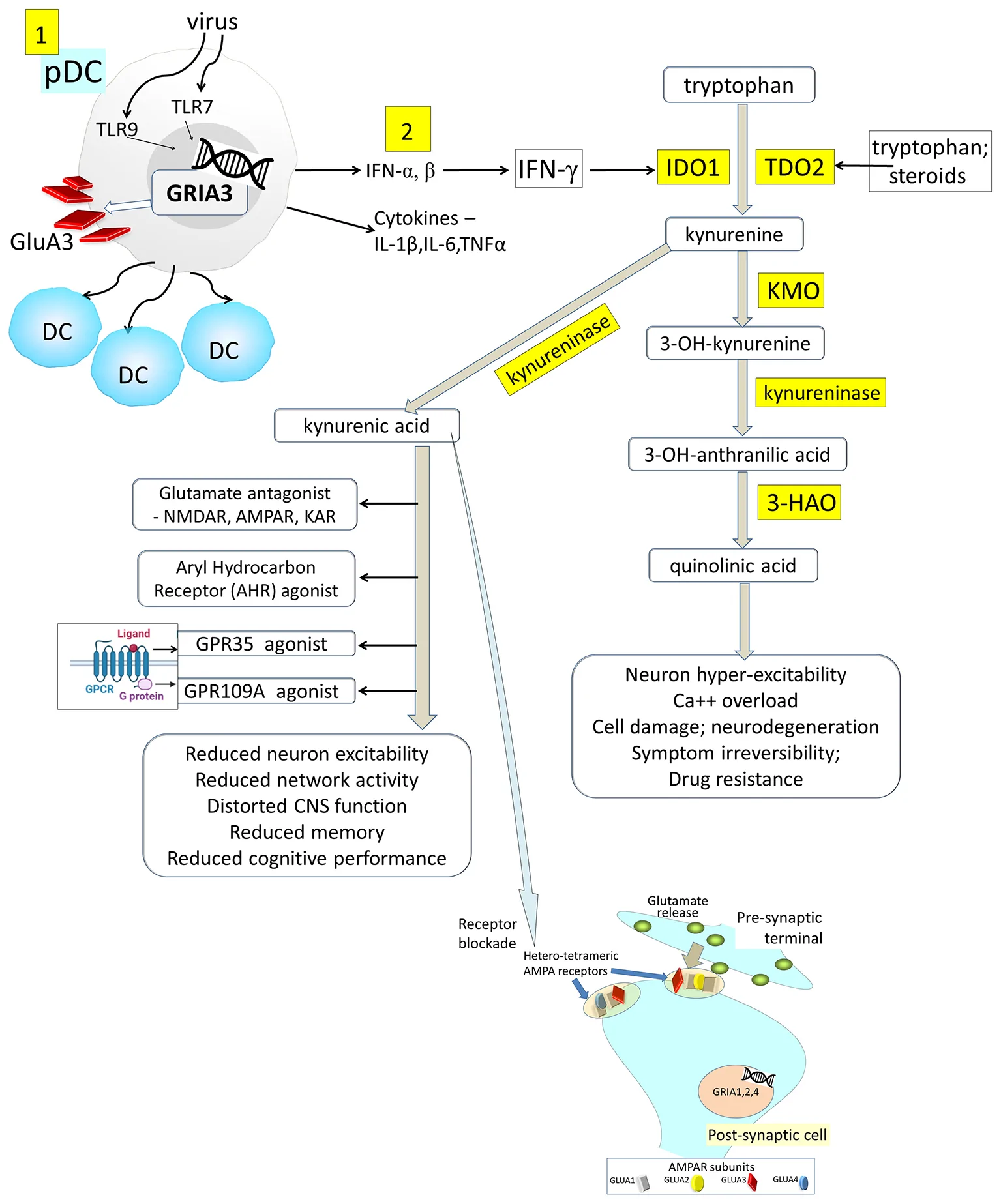
Role of the kynurenine pathway in regulation of the CNS (1) Viral sensing by TLR7 and TLR9 activates pDCs to generate interferons, including IFN-γ (2) which is a potent inducer of IDO1 and the kynurenine pathway (3). Important elements of the pathway include kynurenic acid (4), which blocks AMPAR (5, 6) and activates the AHR and G-protein coupled receptors GPR35 and GPR109A (7). The boxes list the major consequences of kynurenine pathway activity in the CNS.

An additional factor to consider in the context of AMPA receptors is their activation and inactivation, for which it is especially appropriate to consider the kynurenine pathway, the dominant route for tryptophan metabolism (Figure 2). The first enzyme of the pathway is indoleamine-2,3-dioxygenase (IDO1), an enzyme which is powerfully induced by interferon-γ, and whose expression is a key stage in the immune response to challenge, including virus-activated pDCs. Therefore, a viral infection will initiate activity in the kynurenine pathway as a consequence of activating pDCs. The pathway leads to kynurenic acid (Figure 2), an antagonist at all the ionotropic glutamate receptors including AMPAR (activated by quisqualic acid) (Perkins and Stone, 1982; Stone et al., 2013; see Stone, 1993), with additional activity on Aryl Hydrocarbon Receptors (AHR) (Opitz et al., 2011; Coumoul et al., 2026) and G-Protein coupled Receptors such as GPR35 (Berlinguer-Palmini et al., 2013; Resta et al., 2016) and GPR109A proteins (Stone et al., 2022; Stone and Williams, 2024). Kynurenic acid has received much attention as a critical factor in neuroimmune communication because of its regulation of neuronal activity and its promotion of tolerance in the immune system (Stone and Williams, 2023), of which the cross-talk between pDCs and neurons is a specific example. An overview of the kynurenine pathway led to the concept that it operates as an organism-wide “reflex” stress response system. This partly reflects the importance of kynurenic acid acting on AHR to promote differentiation of naïve CD4+ lymphocytes to anti-inflammatory (and anti-autoimmune) Tregs (Huang Y.-S. et al., 2020; Stone and Williams, 2023). This will be amplified by stress-induced glucocorticoids, whether from physical or psychological causes, which activate TDO2, further increasing levels of kynurenic acid in the CNS, where it blocks AMPAR (Figure 2).

Furthermore, the production of TGF-β promotes the expression and activity (enzymatic and non-enzymatic) of IDO1 in IDO1-competent cDCs (Belladonna et al., 2008; Litzenburger et al., 2014) and pDCs (Pallotta et al., 2011; Li et al., 2016), establishing a positive feedback cycle in the differentiation of Tregs in early phases of adaptive immunity with its additional modulation of neuronal excitability and plasticity (Cauwels et al., 2021; Stone and Williams, 2023).

An additional product of the kynurenine pathway is quinolinic acid, a selective agonist at the glutamate receptors sensitive to NMDA (Stone, 1993). Activating the kynurenine pathway, therefore, will produce a complex modulation of network activity in the CNS, depending on the relative concentrations of the kynurenine metabolites and the various subtypes of glutamate receptor (Stone and Williams, 2024).

### 5.1 Kynurenic acid, GluA3, and development

The biological activity of the tryptophan metabolite kynurenic acid was first described using its application to single neurons in anesthetized rats, where it was found to antagonize ligands acting on glutamate receptors. This included blockade of excitation by quisqualic acid, an agonist at the receptors for the later-developed agonist AMPA (Perkins and Stone, 1982; Stone, 1993). Most interest in kynurenic acid has been in its blockade of NMDAR which, as noted above, are a major mediator of neuronal and synaptic plasticity. However, glutamate can only activate NMDARs after initial depolarization by the AMPA and kainate receptors, so these must also be considered as indirect mediators of plasticity, and therefore therapeutic targets comparable with NMDAR.

While the molecular transfer hypothesis could represent one explanation for the development of neuro-psychiatric disorders in the aftermath of physical trauma, infection or chronic stress in individuals of any age (Tioleco et al., 2021; Furman et al., 2019; Solek et al., 2018), it may also be relevant to the disorders initiated by these factors experienced during pregnancy or neonatal life- the so-called “neurodevelopmental disorders.” These are usually considered to be behavioral conditions such as schizophrenia, major depression, and autism spectrum disorders (Jones et al., 2017; Chen et al., 2016; Krakowiak et al., 2012) and the kynurenine pathway represents a potential link between them.

Infection-induced activation of the kynurenine pathway appears to be a key factor affecting CNS development of the embryo and early neonates. This has given rise to the view that some adult disorders are attributable to factors active early in development (Brown, 2011a,b; Meyer et al., 2008; Meyer and Feldon, 2010). Increased levels of kynurenic acid in the CNS of a gestating rodent dam and its embryo results in widespread changes to the structure, biochemistry, and electrophysiological activity in the brains of offspring, persisting into adulthood (Forrest et al., 2013a,b; Khalil et al., 2014; Pisar et al., 2014; Mithaiwala et al., 2021), with associated changes in several aspects of behavior (Buck et al., 2020; Beggiato et al., 2025; Santana-Coelho et al., 2026; Tanaka et al., 2022). This is entirely consistent with extensive evidence from rodents and humans that raised levels of kynurenic acid in the CNS or CSF are related to the early symptoms of schizophrenia (Erhardt et al., 2017; Stone and Darlington, 2013; Kindler et al., 2020; Orhan et al., 2025). Interestingly, the administration of anti-psychotic agents such as clozapine alters *GRIA3* expression in the dorsolateral prefrontal cortex, a region pre-eminently linked with the symptoms of schizophrenia (O'Connor and Hemby, 2007; Duric et al., 2013).

A similar etiology may apply to the cognitive deficits in patients with disorders such as autism and dementias (Huang X. B. et al., 2020; Kegel et al., 2017; Santana-Coelho et al., 2026). The reduced cognitive performance can be prevented by inhibiting the formation of kynurenic acid using inhibitors of KAT. Reducing the synthesis of kynurenic acid from tryptophan and kynurenine using inhibitors of KAT2 is a significant objective of pharmaceutical research (Kozak et al., 2014).

A recent meta-analysis has confirmed the view that chronic stress produces a profound shift in the balance between the neuroprotective activity of kynurenic acid, for which the CNS content is reduced, and the excitatory, potentially neurotoxic activity of quinolinic acid, of which the endogenous concentration is raised (de Bartolomeis et al., 2025). The data would be consistent with a role for elevated kynurenic acid in the early stages of disease, noted above, with a shift toward neurodegeneration following chronic stress and contributing to neurodegeneration and resistance to drug therapy caused by excessive levels of quinolinic acid (Magri et al., 2008). There is also a parallel with the negative correlation between quinolinic acid levels in the blood and the loss of cognitive function in patients with schizophrenia (Cathomas et al., 2022; Stone et al., 2012).

With the strength of this evidence linking kynurenic acid and schizo-affective disorders, and the well-established ability of kynurenic acid to block AMPARs, it is possible that it is the presence of altered AMPAR structures *combined with* the elevated concentrations of kynurenic acid which becomes sufficient to produce the subtle, albeit disabling, disorders of cognition. While the mutations and other molecular irregularities linked to chronic disorders (Table 1) are likely to be a major cause of chronic dysfunction, this combination could increase the functional impact of both factors. A pharmacological strategy which targets kynurenine metabolism in relation to AMPAR structure could be effective in reducing the incidence of several highly drug-resistant disorders.

### 5.2 Kynurenines and pDCs

An additional link may exist between the kynurenine pathway and pDCs. It is been claimed that human pDCs have a high expression of IDO1 which is induced when the pDCs are activated by TLR9 (Chen et al., 2008). Certainly the interferons produced by pDCs are able to increase IDO1 activity (Chalise et al., 2016). This would be valuable since induction of the kynurenine pathway would generate kynurenine and kynurenic acid which initiate the feedback circuit maintaining immune tolerance (Liu et al., 2014; Lippens et al., 2016) via the AHR and Treg differentiation. Not all pDCs may be involved, however, since it has been suggested that the lymph node population express IDO1, whereas splenic pDCs do not (Puccetti and Fallarino, 2008). In the latter population, reverse signaling via IFN-α and IFN-β may be required for IDO1 expression.

## 6 Conclusion

The arguments presented lead to the proposal that plasmacytoid dendritic cells, the leucocyte population which initiates the immune response to viral infections, express an atypical AMPA receptor subunit, namely a flip form of GluA3 with properties that could encourage its transfer to cells in the CNS. The *GRIA3*-GluA3 axis is of fundamental relevance to CNS function, a view supported by the serious medical conditions which result from the loss, mutations or polymorphic variations that have been described for this combination. This would be expected to disrupt normal neuronal circuit activity and lead to the neurological symptoms and disturbed behaviors noted in Table 1. All elements of the proposed links are based on relevant experimental demonstrations and might lead to a consideration of this, or other comparable neuroimmune mechanisms, relevant to understanding the links between infection and disease. The structural stability of AMPARs containing GluA3 in homomeric or heteromeric formations could account for the chronic nature of some illnesses after viral infection. The proposal will require further evidence using specific factors on which the present proposal is based, especially confirmation that *GRIA3* or GluA3 can transfer from the pDCs into CNS cells where their increased expression could contribute to the chronic nature of several disorders. Since the kynurenine pathway is also activated by infection and IFN-γ, changes in the production and concentration of the AMPA antagonist kynurenic acid could contribute to those symptoms.
